# Delayed presentation of an iatrogenic postauricular epidermal inclusion cyst after ear surgery: a case report

**DOI:** 10.1093/jscr/rjaf883

**Published:** 2025-11-11

**Authors:** Shatha Y Alqahtani, Zohour A Almalki, Sara A Assiri, Adnene Moussa, Mohamed Ibrahim

**Affiliations:** Department of Otolaryngology, Alhada Armed Forces Hospital, Prince Mohammed Bin Abdulaziz Road, Taif City 26523, Saudi Arabia; Department of Otolaryngology, Makkah Health Cluster, Al Madinah Al Munawarah Road, Al Aziziyah District, Makkah City 24381, Saudi Arabia; Department of Otolaryngology, King Faisal Medical Complex, Airport Road, Taif City 26523, Saudi Arabia; Anatomic Pathology Division, Alhada Armed Forces Hospital, Prince Mohammed Bin Abdulaziz Road, Taif City 26523, Saudi Arabia; Department of Otolaryngology, Alhada Armed Forces Hospital, Prince Mohammed Bin Abdulaziz Road, Taif City 26523, Saudi Arabia

**Keywords:** postauricular swelling, epidermal inclusion cyst, iatrogenic complication, otologic surgery, head and neck cyst

## Abstract

Epidermal inclusion cysts (EICs) are slow-growing, non-malignant lesions typically arising from entrapped epidermal cells within the dermis. While commonly seen in areas such as the face and neck, their occurrence behind the ear is uncommon. In rare instances, EICs may develop after surgery due to the unintended implantation of skin epithelium during the closure process. We describe a 26-year-old male who developed a gradually enlarging mass in the left postauricular area, 2 years following tympanoplasty. The patient reported no associated symptoms or trauma. The physical examination revealed a firm, tender swelling without any skin changes. Imaging indicated a soft tissue lesion with mastoid sclerosis. Initial antibiotic therapy was ineffective. Surgical exploration revealed a cystic structure, which was completely removed. Histological evaluation confirmed an EIC with no malignant features. The patient recovered without complications. This case illustrates a rare postoperative occurrence of an EIC following ear surgery. Given the potential for such lesions to mimic other conditions, clinicians should maintain a broad differential diagnosis. Proper surgical closure technique may help reduce the risk of epithelial cell entrapment. Complete excision remains the definitive treatment for preventing recurrence and ensuring optimal patient outcomes.

## Introduction

Epidermal inclusion cysts (EICs), also referred to as epidermoid cysts, are non-cancerous subcutaneous lesions composed of keratin and lined by squamous epithelium. They typically develop when epidermal tissue becomes trapped within the dermis. While many EICs arise spontaneously or have congenital origins, their presence in the postauricular area is uncommon. These cysts typically appear as painless, slow-growing swellings in the skin behind the ear [1]. Clinically, epidermoid cysts are present as nodular, fluctuant subcutaneous lesions that may or may not be associated with inflammation [2]. Formation of EICs beneath healed surgical scars can occasionally occur as an iatrogenic outcome, often linked to improper wound edge alignment during closure. Although unexpected, this preventable outcome highlights the importance of meticulous surgical technique [3]. This report presents a 26-year-old male who developed a gradually enlarging postauricular swelling two years after undergoing left tympanoplasty. Radiologic evaluation and intraoperative findings confirmed the lesion to be an EIC, and histopathological analysis ruled out malignancy. This case highlights the importance of including iatrogenic EICs in the differential diagnosis of post-surgical swellings in the head and neck region, underscoring the necessity of meticulous surgical technique and awareness of this rare but possible complication.

## Case presentation

**Figure 1:**
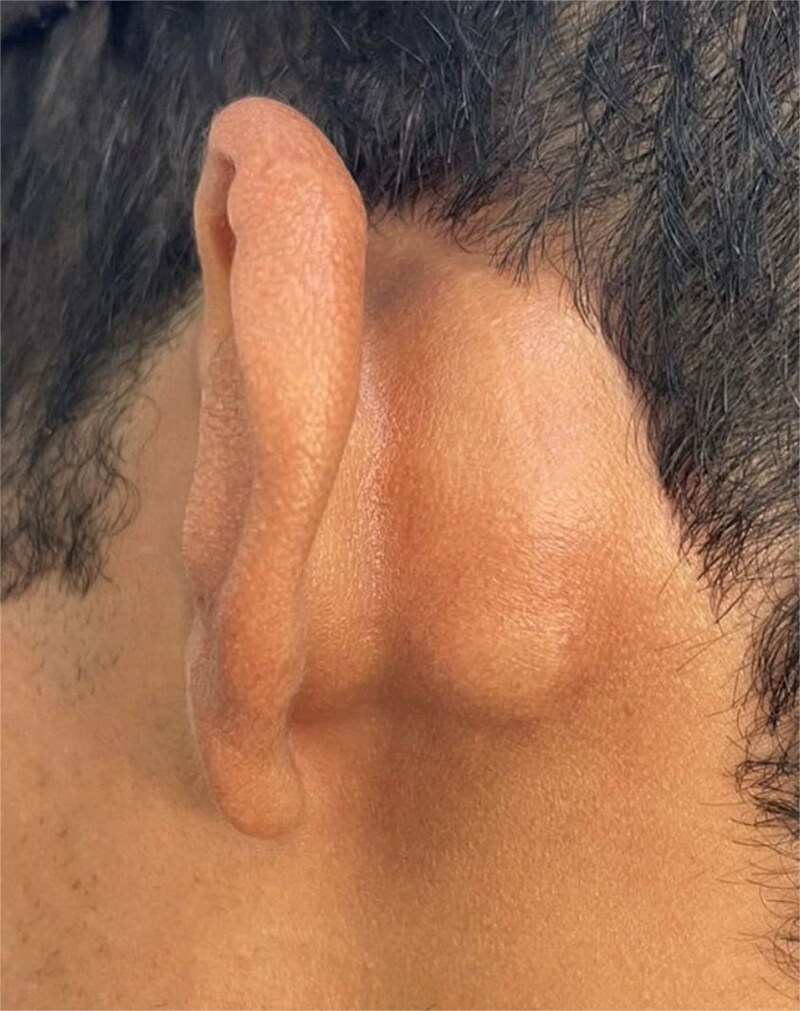
A 3 × 2 cm left postauricular lump, which is cystic and firm on palpation without fluctuation or tenderness after an antibiotic course.

A 26-year-old male with no significant medical history presented to the emergency department with a complaint of left postauricular swelling that had been progressively increasing in size over the past 2 months, with a notable recent increase over the preceding 10 days. The patient denied any associated pain, fever, ear discharge, hearing loss, vertigo, tinnitus, facial asymmetry, or history of trauma. Surgical history was significant for a left tympanoplasty performed 2 years prior at a private hospital, which was complicated by transient facial palsy that resolved spontaneously. The clinical examination revealed a conscious, oriented, and vital patient with bilaterally intact facial nerve function (House-Brackmann Grade I). A firm, tender, non-fluctuant 3 × 2 cm swelling was noted in the left postauricular region without erythema or overlying skin changes (Fig. 1). Otomicroscopy demonstrated normal, intact, and mobile tympanic membranes bilaterally with unremarkable external auditory canals.

**Figure 2:**
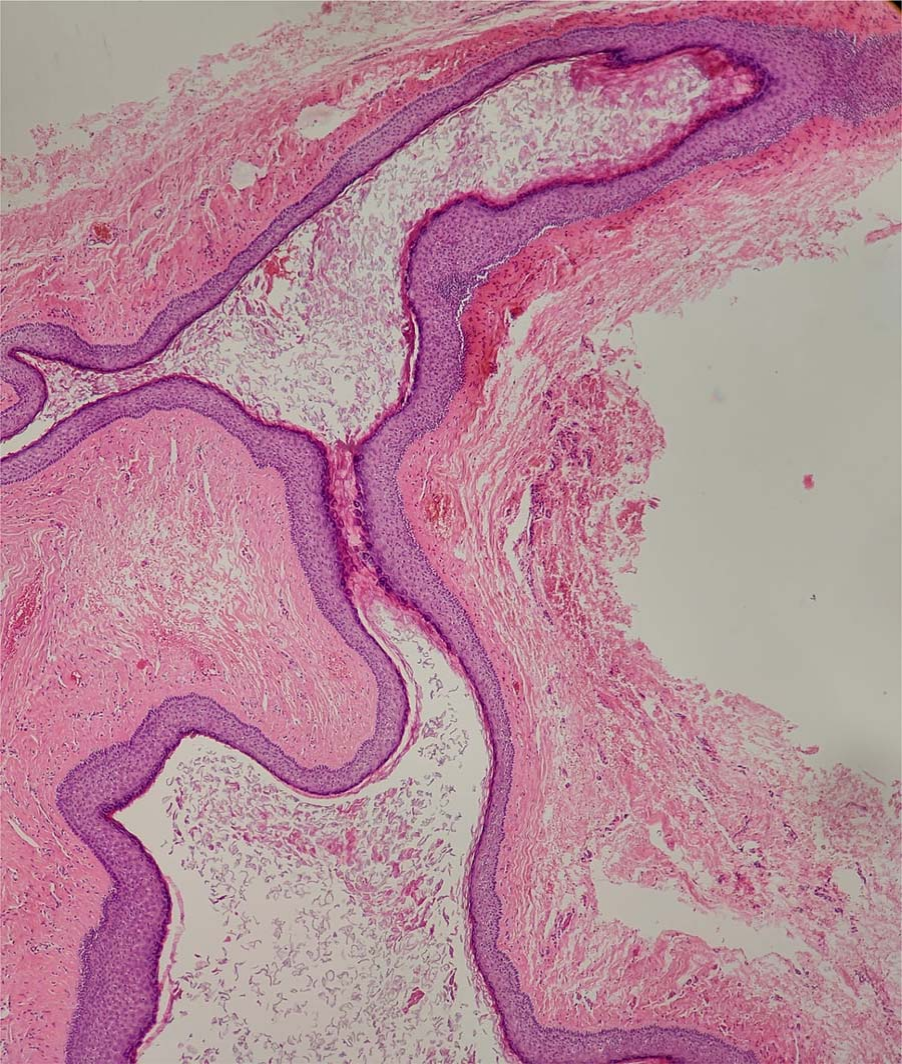
A CT scan reveals a thickened area behind the left ear, indicating the possibility of either a soft tissue abnormality or a cystic lesion.

A non-contrast computed tomography (CT) scan of the head revealed a left retroauricular thickened area suggestive of either soft tissue or cystic lesion, along with left mastoid sclerosis. No definite soft tissue involvement was observed within the middle ear cavity. The ossicles were intact and in normal alignment bilaterally, and the inner ear structures, including the cochlea, semicircular canals, and internal auditory canals, appeared normal (Fig. 2).

**Figure 3:**
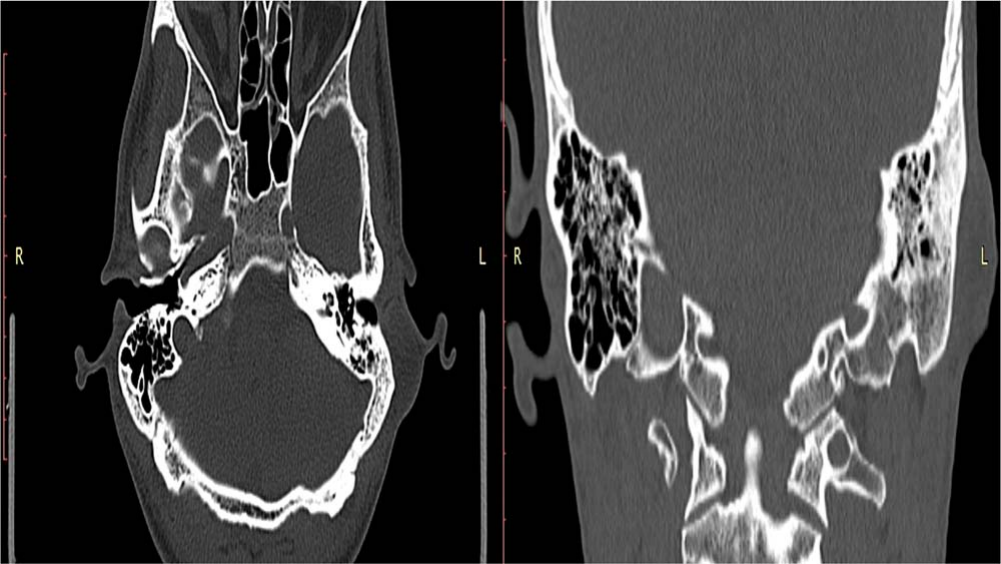
A well-circumscribed cyst lined with stratified squamous epithelium and filled with laminated keratin (hematoxylin and eosin stain, × 40).

The patient was managed initially with a course of oral antibiotics and analgesics and was scheduled for outpatient follow-up. Due to persistent swelling, surgical excision of the lesion was performed under general anesthesia. A longitudinal incision over the previous postauricular scar was made following local infiltration with lidocaine. Intraoperatively, a cystic lesion with a thin capsule was identified; its contents spilled upon dissection, and the entire capsule with its contents was excised. Hemostasis was achieved, and the wound was irrigated with saline and povidone-iodine solution before undergoing layered closure and application of a sterile dressing. Histopathological analysis confirmed the diagnosis of an EIC with no evidence of malignancy. The patient tolerated the procedure well and was discharged in a stable condition with a planned follow-up appointment in the outpatient clinic (Fig. 3).

## Discussion

Postauricular swelling can arise from various etiologies, including congenital anomalies, traumatic lesions, infections, lymphadenopathy, neoplasms, and benign skin masses such as lipomas, sebaceous cysts, or epidermoid cysts. A history of prior surgery or trauma may also contribute to lesion formation in this region, particularly through iatrogenic processes that lead to implantation of epithelial tissue [4].

Although EICs are frequently encountered in dermatological practice, they account for a relatively small proportion of lesions in the head and neck, estimated at around 7%. When present, they are more often located in the periorbital region, face, or neck [5]. These cysts can develop at any age and across various anatomical sites, although they are rarely found in the postauricular area [6]. EICs are histologically classified under dermoid cysts, subdivided into epidermal, dermoid, and teratoid types [5]. The underlying causes of these cysts vary and may involve congenital epidermal cell entrapment, blockage of hair follicles, or implantation of epithelial cells due to trauma or surgical procedures [7].

Procedures involving postauricular incisions in otologic surgery inherently breach skin layers, which can inadvertently introduce epidermal elements into the subcutaneous tissues, creating a potential site for cyst development [8]. Such cysts typically present as slow-growing, non-tender masses; however, inflammation, tenderness, or rupture may occur. Given their indolent nature and location behind the ear, patients often delay medical evaluation until the lesion becomes cosmetically concerning or symptomatic. Although rare, chronic lesions in the head and neck region have been associated with malignant transformation, underscoring the importance of timely diagnosis and management [9].

Radiological imaging plays a crucial role in evaluating postauricular swellings. Ultrasound serves as the initial modality of choice, offering a non-invasive means to assess lesion size, consistency, and vascularity. Advanced imaging, such as CT or magnetic resonance imaging (MRI), may be employed when there is suspicion of deep tissue involvement or when differentiation from other pathologies is necessary. In instances where the facial nerve may be at risk, intraoperative nerve monitoring is advisable to reduce the potential for iatrogenic injury during surgical excision [4, 9].

The treatment of choice for EICs is complete surgical excision, which ensures the total removal of the cyst wall and its contents. Failure to excise the cyst entirely increases the risk of recurrence. Therefore, meticulous dissection and thorough intraoperative inspection are essential components of successful management. Follow-up is equally important to detect any signs of recurrence or complications [4–6].

## Conclusion

Postauricular swelling in a patient with a history of otologic surgery should raise clinical suspicion for iatrogenic EICs. Although these lesions are benign, their development may cause patient distress and can mimic more concerning pathologies. Literature highlights the role of surgical trauma in facilitating epithelial implantation, making EICs a key differential diagnosis in such cases. In our case, the patient underwent successful surgical excision of the lesion, with no immediate complications. Long-term follow-up is planned to monitor for potential recurrence, reaffirming the importance of comprehensive care in similar clinical scenarios.
